# Early right ventricular dysfunction after primary percutaneous coronary intervention in anterior versus isolated inferior myocardial infarction assessed by tissue Doppler imaging and speckle tracking echocardiography

**DOI:** 10.1007/s10741-022-10278-y

**Published:** 2022-10-26

**Authors:** Hanan Ibrahim Radwan, Ahmed Mostafa Abdelhaleem Alhoseeny, Salwa Mohamed Ghoniem, Baher Nabil Eldesouky Nashy, Islam Elsayed Shehata

**Affiliations:** 1 Department of Cardiology, Faculty of Medicine, Zagazig University, Sharkia Governorate, Zagazig, 44519 Egypt; 2 Department of Cardiology, Mansoura Health Insurance Hospital, Sandoub, Mansoura, Dakahlia Governorate Egypt

**Keywords:** Echocardiography, Myocardial infarction, Right ventricle, PCI, STEMI

## Abstract

This study hypothesized that imaging provides information indicating the right ventricular (RV) involvement after anterior or inferior ST-elevation myocardial infarction (STEMI), beyond standard electrocardiogram (ECG) due to the increasing interest in RV function and assessment techniques. This study aimed to compare RV function between anterior and inferior MI without RV involvement using different echocardiographic modalities. This study included 100 patients with anterior (50 patients) and inferior (50 patients) STEMI, who underwent primary percutaneous coronary intervention (PPCI) and two-dimensional echocardiographic imaging within 24 h after PPCI with RV function analysis by left ventricular (LV) infarct size, LV filling pressure, and RV strain rate. Our primary endpoint was the subclinical RV dysfunction in anterior or inferior MI using tissue Doppler and speckle tracking (STE). The study population included 80 (80%) males and 20 (20%) females. Patients with the anterior STEMI had higher mean creatine kinase-MB (CKMB) and troponin than those with inferior STEMI. This study revealed worse RV dysfunction in patients with anterior than those with inferior STEMI, as reflected by significantly lower RV systolic function, tricuspid annular plane systolic excursion (*p* ≤ 0.0001), tissue Doppler-derived velocity (*p* ≤ 0.0001), and STE-derived strain magnitude and rate (*p* ≤ 0.0001). RV dysfunction occurs in patients without ECG evidence of RV STEMI. RV dysfunction is worse in anterior than inferior MI. Moreover, RV systolic functions were affected by declined LV ejection fraction irrespective of the infarction site, which clinically implies prognostic, treatment, survival rate, and outcome improvement between both conditions. (Trial registration ZU-IRB#:4142/26–12-2017 Registered 26 December 2017, email: IRB_123@medicine.zu.edu.eg).

## Introduction

Inferior and anterior myocardial infarctions (MIs) lead to different right ventricular (RV) hemodynamic responses [1].

Two-dimensional (2D) strain and strain rate analyses are novel Doppler-independent approaches to obtain myocardial mobility and deformation measures that are widely employed to assess left ventricular (LV) functions [2]. The subclinical RV dysfunction remained a very important prognostic factor in acute first anterior MI and is not well understood.

The quantitative assessment of RV function remained difficult and is not routinely used in clinical practice because of its complex structure and it is nearly often considered as a reservoir [3]. Cabin et al. [4] studied 97 hearts with anterior MIs, of which 13% were associated with RVMIs.

The quantification of velocity gradients between two sites is possible with strain rate (SR) imaging. The resulting contraction variable does not affect passive tethering effects from other regions; thus, it is a suitable candidate for quantifying regional myocardial function [5].

This study hypothesized that imaging provides RV involvement information after anterior or inferior MI, beyond standard electrocardiogram (ECG). This is linked with the increasing interest in RV function and assessment techniques of what was once often considered a forgotten ventricle.

This study aimed to compare RV function between patients with anterior and inferior MIs without RV involvement using different echocardiographic modalities.

Results may lead to treatment changes and a better understanding of the varying survival rate and different outcomes between both conditions regarding mortality, cardiogenic shock prevalence, and the need for mechanical or inotropic support [6].

## Materials and methods

This single-center comparative cross-sectional study was conducted from January 2018 to January 2020 at the Cardiology Department of our university. The protocol was approved by our University Institutional Review Board which confirmed that all methods were performed following the ethical guidelines of the 1975 Declaration of Helsinki as reflected in a priori approval by the institution’s human research. Informed written consent was obtained from all participants.

Patients were prospectively recruited. The study group comprised a convenience sample of 100 consecutive age, sex, RV systolic pressure, and pharmacological therapies administered before the percutaneous coronary intervention (PCI)-matched subjects admitted to our critical care unit with the first episode of acute STEMI according to the STEMI guidelines [7] and referred for primary PCI (PPCI) who fulfilled the inclusion criteria. The onset of chest pain was < 3 h. The time interval between onset of chest pain and PPCI was 1–3 h and transthoracic echocardiography (TTE) was done within 24 h after PPCI. The subjects were divided into two groups following ECG recording. Group I (anterior MI) included 50 patients with ST-elevation in the anterior and lateral leads (V1–V6, aVL, and I) and group II (isolated inferior MI) included 50 patients with ST-elevation in the inferior leads (II, III, and aVF).

### Inclusion criteria

This study included 100 patients with first anterior and inferior STEMI who underwent PPCI. Acute STEMI was defined as evidence of ischemic chest pain for > 30 min and new ST-segment elevation in two or more contiguous ECG leads and elevated cardiac troponin. Anterior MI was defined as ST-segment elevation of at least 1 mm in anterior leads (V1–V6, aVL, and I). Inferior MI was defined as ST-segment elevation of at least 1 mm in inferior leads (leads II, III, and aVF) [7].

### Exclusion criteria

ST-elevation in V_3_R, V_4_R (RV infarction), bundle branch block, or any other interventricular conduction delay, MI history, previous revascularization, rheumatic heart disease, cardiomyopathy, cardiogenic shock, acute pulmonary congestion, heart failure, previous documented abnormal LV function, rhythm disturbance, pulmonary hypertension, pulmonary embolism, liver cell failure, chronic renal failure, hemodynamic instability, and poor echogenic window.

### Study methodology

All patients were subjected to *history taking* and a *thorough physical examination*, including pulse and systolic and diastolic blood pressure measurements.

#### Resting 12-lead standard surface electrocardiogram 

upon admission and ST-segment elevation were analyzed according to the joint European Society of Cardiology, American College of Cardiology (ACC), American Heart Association (AHA), and World Heart Federation task force for the universal MI definition [8]. Disproportionate ST-segment elevation with greater ST-elevation in lead III than in lead II is pathognomonic for an RV MI, and RV involvement should be considered [9]. A sole ST-segment elevation in lead V4R of > 1.0 mm is a reliable RVMI indicator, with 100% sensitivity, 87% specificity, and 92% predictive accuracy [10]. Furthermore, higher ST-segment elevations in V4R were independent predictive factors for more significant RV dysfunction and higher mortality rates [11].

#### Laboratory investigations

with special emphasis on cardiac markers (troponin and CKMB) and creatinine.

#### TTE

**Fig. 1:**
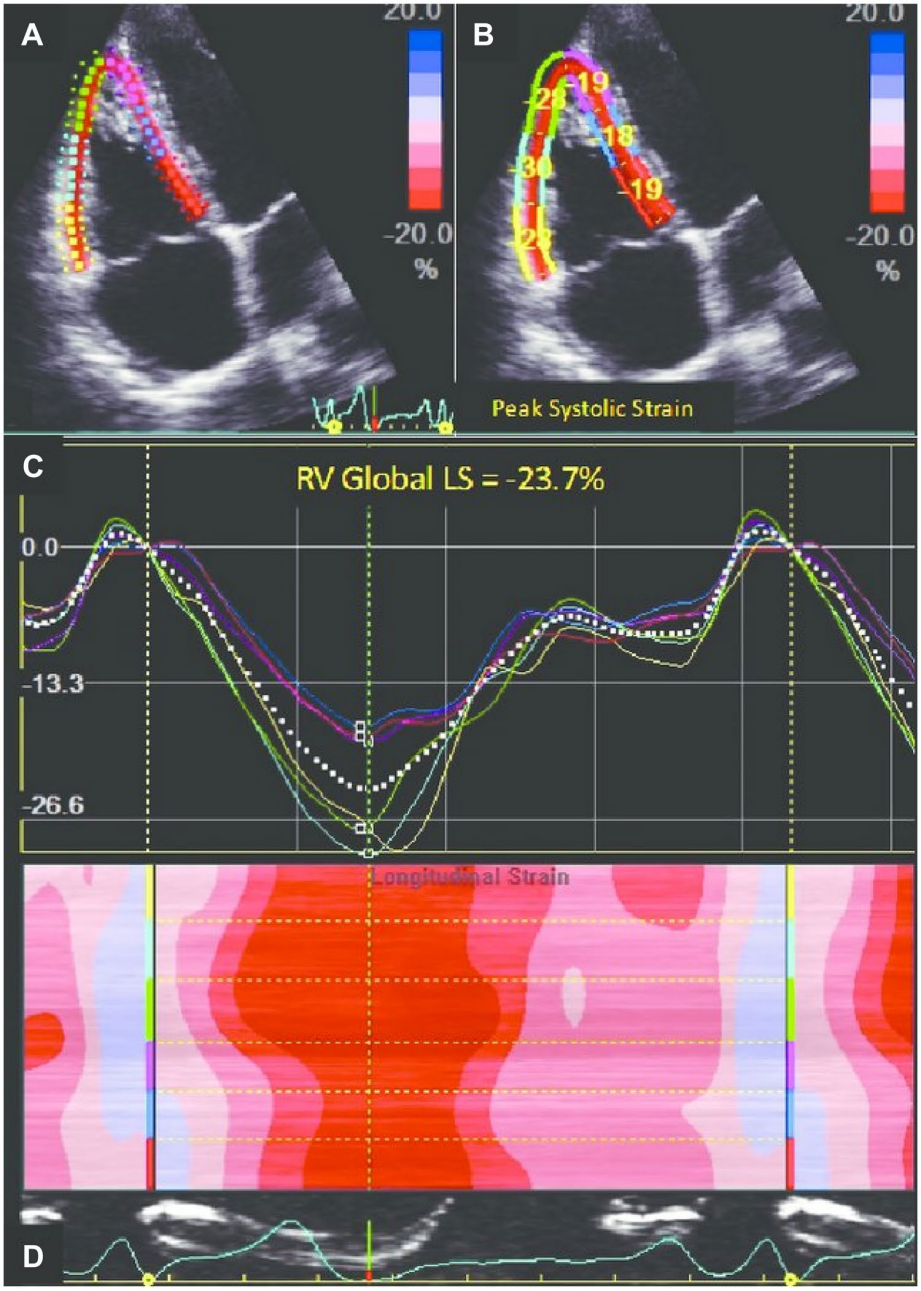
Peak systolic longitudinal strain of the right ventricular (RV) free wall and interventricular septum obtained with two-dimensional speckle tracking analysis; **A** Parametric color-coded display of end-systolic strain; **B** Regional end-systolic strain; **C** Strain–time curves. Colored curves show the segmental strain change during the cardiac cycle, and the white dotted line shows the global RV strain changes during the cardiac cycle; **D** Anatomical M-mode color-coded display of segmental strain variations during the cardiac cycle (global longitudinal strain [GLS] =  − 23.7%)

All participants underwent conventional TTE within 24 h after PPCI using the commercially available system (Vivid 9, GE Health care, Waukesha, WI, USA, 2013). Data acquisition was done by 2 independent operators and the mean values were interpreted to avoid LAB inter- and intra-observer variability. Data acquisition was performed at a depth of 16 cm in the parasternal long axis, apical 4 and apical 2 chamber views, using a 1.5–4.3-MHz M4S transducer. During hold-breath, M-mode and 2-D images were obtained in 3 consecutive beats and were saved. The reference limits of all recorded and calculated parameters were defined according to the American Society of Echocardiography Guidelines [12]. *Left ventricular assessment included* LV ejection fraction (EF%) measurement by modified Simpson’s method and LV fractional shortening (FS%) to evaluate LV systolic function and LV volume. Pulsed wave Doppler (PW) echocardiography was used to evaluate LV diastolic function. The peak E wave velocity, the peak A wave velocity, and the *E*/*A* ratio were measured by conventional PW Doppler. The peak e′ wave velocity, the peak a′ wave velocity, and *E*/*e*′ ratio were measured by tissue Doppler imaging (TDI); the assessment of LV infarct size depends on EF and LV filling pressure parameters. *RV assessment included* 2D and tissue Doppler; RV minor dimension at the basal ventricular level measured in end-diastole; right atrial (RA) minor dimension measured in end-systole; tricuspid annular plane systolic excursion (TAPSE); myocardial performance index (MPI) by pulsed wave Doppler method; pulsed wave tissue Doppler imaging (PW-TDI); pulsed wave Doppler imaging (PW); and strain, SR, and speckle tracking (Fig. 1).

#### Coronary angiography and primary PCI

Coronary angiography was performed using the percutaneous radial approach by the Sildenger technique. Right and left coronary angiography was performed using multiple projections, and analysis was done by professional interventionists who were blind to the cases. Drug-eluting stents, or more specifically everolimus-eluting stents, were implanted using a routine method, and procedure success indicated residual stenosis of < 20%, thrombolysis in MI (TIMI) flow grade III, and no in-hospital acute complications (death, myocardial infarction, and emergency coronary artery bypass grafting [CABG]), and no major adverse cardiac events (cardiac death, myocardial infarction, and target vessel revascularization) [13]. All patients underwent PCI for the culprit and non-culprit lesions with successful TIMI III flow results with no residual coronary stenosis for all studied patients. Lesions were classified according to the ACC/AHA classification based on the morphological characteristics of lesions that cause coronary artery stenosis [14]. All clinical, laboratory, and coronary angiographic data were evaluated by two independent investigators who were not involved in the angiographic procedures.

Our primary endpoint was to assess the subclinical RV dysfunction in anterior or inferior MI using tissue Doppler and speckle tracking.

### Statistical analysis

All data were analyzed using IBM Statistical Package for the Social Sciences software version 23 for windows (SPSS, Inc. Chicago, IL, USA). The statistical analysis was conducted following the principles as specified in the International Council for Harmonization Topic E9 (ICH1998). Results were presented as mean value ± standard deviation for continuous variables and as frequency (%) for categorical variables. Data were tested for normality using the Kolmogorov–Smirnov test. The following tests were used to test differences for significance: chi-square test (*X*^2^) for qualitative variable, *t*-test for quantitative independent groups, and correlation coefficient for testing association between two variables. A *p*-value of ≤ 0.05 was considered a significant difference for all statistical analyses.

## Results

Our study population included 80 (80%) males and 20 (20%) females divided into 2 groups. There was no significant difference in the median age between the anterior and inferior STEMI groups (*p* = 0.793). Our study showed no statistically significant differences between both groups regarding demographic data (Table 1).

**Table 1:** Clinical characteristics of the studied groups

		**Anterior MI group** **(*N* = 50)**	**Inferior MI group** **(*N* = 50)**	***p*-value**
**Age (years)**		**62.56 ± 19.94**	**61.8 ± 4.47**	**0.793**^**NS**^
**Weight (kg)**		**101.16 ± 10.4**	**97.24 ± 15.25**	**0.137**^**NS**^
**Height (cm)**		**165.62 ± 9.19**	**164.31 ± 8.58**	**0.076**^**NS**^
**BMI (kg/m**^**2**^**)**		**26.53 ± 4.13**	**26.45 ± 5.53**	**0.94**^**NS**^
**Sex**	**Female (*N*&%)** **Male (*N*&%)**	**10 (20%)**	**10 (20%)**	**>0.99**^**NS**^
		**40 (80%)**	**40 (80%)**	
**HTN**	***N* &%**	**25 (50%)**	**21 (42%)**	**0.42**^**NS**^
**DM**	***N* &%**	**25 (50%)**	**21 (42%)**	**0.42**^**NS**^
**Dyslipidemia**	***N* &%**	**28 (56%)**	**25 (50%)**	**0.54**^**NS**^
**Smoker**	***N* &%**	**28 (56%)**	**33 (66%)**	**0.305**^**NS**^
**SBP (mmHg)**		**120.2 ± 8.91**	**119.0 ± 7.69**	**0.473**^**NS**^
**DBP (mmHg)**		**76.2 ± 8.66**	**75.8 ± 8.35**	**0.815**^**NS**^
**HR (bpm)**		**80.48 ± 8.99**	**79.68 ± 10.77**	**0.688**^**NS**^

*DBP* diastolic blood pressure, *DM* diabetes mellitus, *HR* heart rate, *HTN* hypertension, *MI* myocardial infarction, *SBP* systolic blood pressure, *NS* (*p*-value > 0.05) non-significant

Patients with anterior STEMI had significantly higher mean CKMB and troponin than those with inferior STEMI (*p* = 0.012).

Patients with anterior MI had significantly lower LVEF and more diastolic dysfunction in the form of lower mitral septal *E*′ with high filling pressure as they had a significant increase in *E*/*E*′ ratio, which indicates larger infarct size than those with inferior MI (Table 2).

**Table 2:** Left side echocardiographic findings

	**Anterior MI group** **(*N* = 50)**	**Inferior MI group** **(*N* = 50)**	***p-*value**
**LVEF by Simpson (%)**	**38.44 ± 7.66**	**48.54 ± 8.62**	<0.0001**
**LVESD 2D (mm)**	**33.98 ± 3.57**	**31.82 ± 3.57**	0.019*
**LVEDD 2D (mm)**	**55.66 ± 4.87**	**52.62 ± 4.07**	0.006*
**LV mass (g)**	**87.88 ± 16.09**	**88.74 ± 10.22**	**0.752**^**NS**^
**Mitral** ***E/A***	**1.04 ± 0.28**	**0.99 ± 0.18**	**0.281**^**NS**^
**Mitral septal** ***E'*** **(cm/s)**	**7.18 ± 2.66**	**8.24 ± 2.29**	0.036*
**Mitral septal** ***E/E′***	**17.68 ± 2.04**	**14.92 ± 2.29**	< 0.001**

*LV* left ventricle, *LVEDD* left ventricular end-diastolic diameter, *LVEF* left ventricular ejection fraction, *LVESD* left ventricular end-systolic diameter, *NS* (*p*-value > 0.05) non-significant; *significant (*p* < 0.05); **highly significant (*p* < 0.001)

Patients with anterior MI had worse RV systolic function in the form of lower TAPSE and lateral wall systolic velocity (*S*′) and higher MPI of the RV by pulsed (RMPI-PD) and tissue Doppler (RMPI-TDI), as well as worse diastolic function in the form of lower lateral *E*′ velocity, compared to those with inferior MI (Table 3).

**Table 3:** Right side echocardiographic findings

	**Anterior MI group** **(*N* = 50)**	**Inferior MI group** **(*N* = 50)**	***p*-value**
**Right atrial diameter 2D (mm)**	**26.2 ± 8.6**	**24.5 ± 10.4**	0.003*
**Right ventricular diameter 2D (mm)**	**37.86 ± 24.36**	**36.98 ± 25.65**	**0.111**^**NS**^
**TAPSE (mm)**	**15.42 ± 5.7**	**18.16 ± 8.0**	<0.0001**
**Tricuspid E/A**	**1.04 ± 0.19**	**0.977 ± 0.16**	**0.096**^**NS**^
**RMPI PWD**	**0.39 ± 0.07**	**0.32 ± 0.11**	<0.0001**
**PASP mean (mmHg)**	**22.88 ± 5.38**	**19.66 ± 6.78**	0.01*
**PASP max (mmHg)**	**32.28 ± 5.36**	**30.1 ± 7.18**	**0.089**^**NS**^
**Tricuspid lateral** ***E/e′***	**8.0 ± 1.0**	**6.1 ± 1.19**	**0.185**^**NS**^
**Tricuspid lateral** ***E′*** **(cm/s)**	**7.06 ± 2.39**	**8.13 ± 1.96**	0.005*
**Tricuspid lateral** ***S′*** **(cm/s)**	**8.60 ± 1.96**	**10.99 ± 1.78**	0.008*
**RMPI_TDI**	**0.52 ± 0.088**	**0.46 ± 0.105**	0.006*
**IVC_Diameter_2D (mm)**	**16.14 ± 2.91**	**14.1 ± 1.41**	<0.0001**

*IVC* inferior vena cava, *PASP* pulmonary artery systolic pressure *PWD* pulsed wave Doppler, *RMPI* right ventricular myocardial performance index, *TAPSE* tricuspid annular plane systolic excursion, *TDI* tissue Doppler imaging *NS* (*p*-value > 0.05) non-significant; *significant (*p* < 0.05); **highly significant (*p* < 0.001)

Patients with anterior MI had worse RV systolic function compared to those with inferior MI, as evidenced by numerically lower RV strain and lower SR by TDI (Table 4).

**Table 4:** RV free wall strain and strain rate by TDI

	**Anterior MI group** **(*N* = 50)**	**Inferior MI group** **(*N* = 50)**	***p*-value**
**Basal strain (%)**	** − 21.36 ± 3.33**	** − 25.15 ± 5.71**	< 0.0001**
**Mid strain (%)**	** − 17.16 ± 3.34**	** − 21.06 ± 5.69**	< 0.0001**
**Apical strain (%)**	** − 11.47 ± 3.32**	** − 15.51 ± 5.68**	< 0.0001**
**Mean strain (%)**	** − 16.66 ± 3.34**	** − 20.58 ± 5.69**	< 0.0001**
**Basal strain rate (s**^−1^**)**	** − 1.89 ± 0.51**	** − 1.32 ± 0.31**	< 0.0001**
**Mid strain rate (s**^−1^**)**	** − 1.59 ± 0.51**	** − 1.035 ± 0.29**	< 0.0001**
**Apical strain rate(s**^−1^**)**	** − 1.34 ± 0.42**	** − 0.76 ± 0.23**	< 0.0001**
**Mean strain rate (s**^−1^**)**	** − 1.61 ± 0.47**	** − 1.04 ± 0.28**	< 0.0001**

**highly significant (*p* < 0.001)

Patients with anterior MI had worse RV systolic function compared to those with inferior MI, as evidenced by numerically lower RV free wall systolic strain and lower RV global longitudinal strain by speckle tracking (Table 5).

**Table 5:** RV Strain by speckle tracking

**(%)**	**Anterior MI group** **(*N* = 50)**	**Inferior MI group** **(*N* = 50)**	***p*-value**
**Basal septum**	** − 14.92 ± 2.91**	** − 17.12 ± 2.94**	<0.0001**
**Mid septum**	** − 13.92 ± 2.91**	** − 15.32 ± 2.91**	0.018*
**Apical septum**	** − 12.20 ± 3.12**	** − 12.60 ± 2.87**	**0.508**^**NS**^
**Basal free wall**	** − 21.86 ± 3.33**	** − 26.08 ± 5.71**	<0.0001**
**Mid free wall**	** − 18.91 ± 3.33**	** − 22.56 ± 5.69**	<0.0001**
**Apical free wall**	** − 12.46 ± 3.52**	** − 16.51 ± 5.26**	<0.0001**
**Free wall GLS**	** − 17.58 ± 3.38**	** − 21.71 ± 5.26**	<0.0001**
**GLS**	** − 15.62 ± 2.34**	** − 17.48 ± 4.35**	0.009*

*GLS* global longitudinal strain, *NS* (*p*-value > 0.05) non-significant; *significant (*p* < 0.05); **highly significant (*p* < 0.001)

The anterior MI group was significantly associated with the left anterior descending artery (LAD) as the culprit in contrast to the inferior MI group, which was significantly associated with the right coronary artery (RCA) as the culprit (*p* < 0.001).

Regarding significantly vascular stenosis in the anterior MI group, LAD was affected in 50 (100%) patients, left circumflex artery (LCX) in 12 (24%), and RCA in 5 (10%). In the inferior MI group, LAD was affected in 4 (8%) patients, LCX in 6 (12%), and RCA in 50% of patients. Regarding the number of vessels, the anterior MI group had 34 (68%) patients with single-vessel disease, 15 (30%) had two-vessel diseases, and 1 (2%) had three-vessel diseases, whereas the inferior MI group had 42 (84%) patients with single-vessel diseases, 7 (14%) two-vessel diseases, and 1 (2%) with three-vessel diseases. No statistically significant differences were found between both groups regarding the number of affected vessels (Table 6).

**Table 6:** Coronary angiography findings distribution between groups

			**Group**		**Total**	***p*-values**
			**Anterior MI group** **(*N* = 50)**	**Inferior MI group** **(*N* = 50)**		
**Culprit**	**LAD**	***N***	**47**	**1**	**48**	
		**%**	**94.0%**	**2.0%**	**48.0%**	
	**LCX**	***N***	**3**	**0**	**3**	<0.0001**
		**%**	**6.0%**	**0.0%**	**3.0%**	
	**RCA**	***N***	**0**	**49**	**49**	
		**%**	**0.0%**	**98.0%**	**49.0%**	
**Vessels with > 70% stenotic lesion**	**LAD**	***N***	**50**	**4**	**54**	<0.0001**
		**%**	**100.0%**	**8.0%**	**54.0%**	
	**LCX**	***N***	**12**	**6**	**18**	**0.11**^**NS**^
		**%**	**24.0%**	**12.0%**	**18.0%**	
	**RCA**	***N***	**5**	**50**	**55**	<0.0001**
		**%**	**10.0%**	**100.0%**	**55.0%**	
**Number vessels**	**1.00**	***N***	**34**	**42**	**76**	
		**%**	**68.0%**	**84.0%**	**76.0%**	
	**2.00**	***N***	**15**	**7**	**22**	**0.15**^**NS**^
		**%**	**30.0%**	**14.0%**	**22.0%**	
	**3.00**	***N***	**1**	**1**	**2**	
		**%**	**2.0%**	**2.0%**	**2.0%**	

*LAD* left anterior descending artery, *LCX* left circumflex artery, *RCA* right coronary artery, *NS* (*p*-value > 0.05) non-significant; **highly significant (*p* < 0.001)

Table 7 shows a significant positive correlation between EF and TAPSE in the anterior and inferior MI groups. EF showed a significant negative correlation with free wall global longitudinal strain (GLS) and GLS in both groups. EF showed a significant negative correlation with MPI by PW and MPI by TDI in both groups.

**Table 7:** Correlation between ejection fraction and right ventricular systolic functions

**Group**			**EF**
**Anterior MI group**	**TAPSE**	**R**	0.296*
		**P**	**0.037**
	**Basal free wall**	**R**	**−0.031–**
		**P**	**0.830**^**NS**^
	**Mid free wall**	**R**	**−0.029–**
		**P**	**0.844**^**NS**^
	**Apical free wall**	**R**	**−0.058–**
		**P**	**0.692**^**NS**^
	**Free wall GLS**	**r**	−0.332–*
		**P**	**0.021**
	**GLS**	**r**	−0.298*
		**P**	**0.036**
	**MPI (conventional)**	**r**	−0.312–*
		**P**	**0.014**
	**MPI (tissue Doppler)**	**r**	−0.374–*
		**P**	**0.011**
**Inferior MI group**	**TAPSE**	**r**	0.467**
		**P**	**0.001**
	**Basal free wall**	**r**	**−0.187–**
		**P**	**0.195**^**NS**^
	**Mid free wall**	**r**	**−0.212–**
		**P**	**0.140**^**NS**^
	**Apical free wall**	**r**	**−0.207–**
		**P**	**0.150**^**NS**^
	**Free wall GLS**	**r**	−0.317*
		**P**	**0.025**
	**GLS**	**r**	−0.294*
		**P**	**0.036**
	**MPI (conventional)**	**r**	−0.295–*
		**P**	**0.035**
	**MPI (tissue Doppler)**	**r**	−0.325–*
		**P**	**0.012**

*MI* myocardial infarction, *TAPSE* tricuspid annular plane systolic excursion, *GLS* global longitudinal strain, *MPI* myocardial performance index, *NS* (*p*-value > 0.05) non-significant; *significant (*p* < 0.05); **highly significant (*p* < 0.001)

## Discussion

From the physiological point of view, given the distribution of the perfusion territories of the major epicardial coronary arteries, inferior MI in most cases is due to RCA stenosis, which supplies both the RV free wall and the base of the interventricular septum and would thus affect most RV functional measurements while anterior MI is usually due to LAD artery stenosis, which does not significantly contribute to RV perfusion other than the RV apex when the LAD artery wraps around the apex [15]. Hence, RV dysfunction is more common in inferior LV infarction due to similar vascularization. However, the opposite occurs. The lower EF in the anterior MI group may drive the results finding of the association between RV dysfunction and anterior MI. EF was independently correlated to the RV dysfunction parameter of the LV infarction site. Our study revealed more RV dysfunction in anterior MI than inferior MI due to the synchronous LV and RV mechanical dysfunction based on the Torrent-Guasp model. Presumably, patients with anterior MI had an overall worse myocardial involvement as demonstrated by the worse LVEF and left ventricular end-diastolic diameter (LVEDD). Therefore, as demonstrated by previous studies, RV involvement is more present in the first group as it is representative of a more extensive disease. Moreover, a significant percentage of patients with non-culprit lesions with severe stenosis was found in both groups (20% vs. 34% in the inferior vs. anterior MI group, respectively, *p* < 0.0001), which may be a confounding factor in RV function evaluation.

LV systolic function and LVEF by Simpson’s method were highly significantly lower in the anterior MI group compared to the inferior MI group (*p* < 0.0001). Additionally, LV filling pressure was significantly higher in the anterior MI group (*p* < 0.001). This data was concordant with Abtahi et al. [15] who compared RV function in 60 patients, including 25 in group 1 and 35 in group 2 (acute myocardial infarction). Synchronous LV and RV mechanical dysfunction may be explained by the Torrent-Guasp model as the helical ventricular myocardial band encircles the right and left ventricles with transversely oriented circular myocardial fibers within a basal loop, while intermingled with two differently oriented oblique fibers, directed from right to left within the sub-endocardial muscle and from left to right within the sub-epicardial muscle, crisscross within the interventricular septum and connect in the apex of the heart. The cross-striation of oblique fibers in the ventricular septum increases septal twisting motion, which benefits both RV and LV performance [16].

LVEDD was significantly higher in the anterior MI group compared to the inferior MI group although both were within the normal range (*p* < 0.05). These data were concordant with Korup et al. [17] who examined 35 consecutive patients who were admitted to the hospital with the first episode of anterior or inferior STEMI and revealed increased end-systolic and end-diastolic measurements as early as 3 h after onset of symptoms in both groups, although LV dilatation was more pronounced in the anterior MI group. Slippage of necrotic myofibrils causing stretching of the infarct zone is caused by early LV dilatation.

RA and RV dimensions and RA diameter were significantly higher in the anterior MI group compared to the inferior MI group (*p* < 0.001). RV diameter was significantly higher in the anterior MI group compared to the inferior MI group although RV diameter was higher than the normal range in both groups (*p* < 0.05). These data were discordant with Abtahi et al. [15] who observed no significant difference between patients with anterior MI and inferior MI regarding RV and RA diameters. This could be explained by more RV involvement in the first group as it is representative of a more extensive disease. Moreover, a significant percentage of patients with non-culprit lesions had severe stenosis (20% vs. 34% in the inferior vs. anterior MI groups, respectively, *p* < 0.0001).

RV systolic function and TAPSE were significantly lower in the anterior MI group compared to the inferior MI group (*p* < 0.05). TAPSE was below the normal value in the anterior MI group while within the normal range in the inferior MI group. These data are concordant with Keskin et al. [18] who studied RV functions in 350 patients with first-time anterior STEMI and revealed significant RV systolic function reduction, including TAPSE. The TAPSE reduction in the anterior STEMI group is explained by the positive correlation between TAPSE and LVEF regardless of the RV systolic functions. Thus, the concept of ventricular interdependence shown in experimental models is an important explanatory factor in the relationship between TAPSE and LVEF.

MPI of RV (RMPI) by pulsed and tissue Doppler was significantly higher in the anterior MI group and normal in the inferior MI group with a significant difference between both groups (by PW, *p* = 0.00; by TDI, *p* = 0.006). These data are concordant with Ozturk et al. [19] who revealed a significant increase in this index after anterior STEMI.

The systolic velocity of the lateral wall of RV by tissue Doppler (*S*′) was significantly lower in the anterior MI group compared to the inferior MI group (*p* = 0.008). These data are concordant with Hsu et al. [20] who revealed lower RV annular velocities, including *S*′ in the anterior STEMI group compared to the inferior STEMI group. Hsu et al. [20] revealed that RV systolic functions might be affected regardless of the infarction site in patients with a first acute ST-elevation MI not associated with RV infarction, in agreement with our findings regarding RV systolic functions. Furthermore, he noted a depressed global systolic RV function in those with anterior MI.

RV systolic functions have a multitude of factors and are strongly dependent on LV function and shape and septal motion. Additionally, the unique overlapping nature of territories of coronary arteries and the variable patterns between different patients can explain these correlations.

RV diastolic function and tricuspid *E*/*A* ratio were normal in both groups with no significant difference (*p* = 0.096). However, lateral *E*′ was significantly lower in the anterior MI group compared to the inferior MI group (*p* = 0.005) while no significant difference between both groups regarding lateral *E*/*e*′ (*p* = 0.185). This data is in disconcordant with the findings of Hsu et al. [20], where RV diastolic dysfunction was reported in patients with inferior infarction, whereas Abtahi et al. [15] reported no significant differences between both groups regarding RV diastolic functions.

The tricuspid *E*/*E*′ ratio and diastolic SR are commonly utilized to assess RV diastolic function.

Demirkol et al. [21] revealed a substantial link between the tricuspid *E*/*e*′ ratio, RA volume, and hemodynamic parameters in the tricuspid valve. The tricuspid *E*/*E*′ ratio predicts cardiac events and increased mean arterial pressure as a reliable measure of RV filling pressure. An *E*/*e*′ ratio of > 6 exhibited good sensitivity and specificity in identifying the mean RA pressure of 10 mmHg.

The systolic velocity of the lateral wall of RV, as assessed by tissue Doppler (*S*′), was significantly lower in the anterior MI group compared to the inferior MI group (*p* = 0.008). These data are concordant with Hsu et al. [20] who reported lower RV annular velocities, including *S*′ in the anterior STEMI group compared to the inferior STEMI group. Hsu et al. [20] revealed that RV systolic functions might be affected regardless of the infarction site in patients with a first acute STEMI not associated with RV infarction, in agreement with our findings regarding RV systolic functions. Furthermore, he noted depressed global systolic RV function in patients with anterior infarction.

Concerning the RV diastolic function, the tricuspid *E*/*A* ratio was normal in both groups with no significant difference (*p* = 0.096). However, lateral *E*′ was significantly lower in the anterior MI group compared to the inferior MI group (*p* = 0.005) while no significant difference between both groups regarding lateral *E*/*e*′ (*p* = 0.185). These data are discordant with the findings of Hsu et al. [20], where RV diastolic dysfunction was reported in patients with inferior MI, whereas Abtahi et al. [19] reported no significant differences between both groups regarding RV diastolic functions. This could be explained by a significant percentage of patients with non-culprit lesions with severe stenosis (20% vs 34% in the inferior vs. anterior MI groups, respectively, *p* < 0.0001).

RV free wall tissue velocity was lower than the average with no significant difference between both groups. RV strain was significantly lower in the anterior MI group compared to the inferior MI group (*p* < 0.001). RV septal strain was significantly lower in the anterior MI group compared to the inferior MI group (*p* < 0.001 and *p* = 0.018, respectively), as well as the RV free wall strain (*p* < 0.001). RV-GLS was significantly lower in the anterior MI group compared to the inferior MI group (*p* = 0.009).

RV systolic function using strain assessed by TDI and speckle tracking is disconcordant with that of Huttin et al. [22] who demonstrated reduced RV strain values in all MI sites; however, it was more pronounced in inferior than anterior MI. In contrast, the septal strain was the same in both groups. Notably, Huttin et al. [22] did not exclude patients with electrocardiographic evidence of RV STEMI.

Mohamed et al. [23] excluded patients with electrocardiographic evidence of RV infarction and enrolled 80 patients with anterior STEMI who were subjected to PPCI. RV functions were assessed using strain by speckle tracking, which revealed a significant relationship between RV affection and anterior STEMI, which was consistent with our findings.

Nourian et al. [24] evaluated RV systolic and diastolic functions between inferior STEMI without RV involvement and inferior STEMI with RV infarction and revealed that the RV-GLS in the inferior STEMI group without RV infarction was normal, which is in concordant with our findings.

Our results showed a significant positive correlation between EF and TAPSE in the anterior (*r* = 0.296, *p* = 0.37) and inferior MI groups (*r* = 0.467, *p* = 0.001). Antoni et al. [25] revealed a substantial positive correlation between LVEF and TAPSE, regardless of the site of the STEMI in patients with acute STEMI, similar to our findings.

## Study limitations

Our single-center analysis included a relatively small sample size; thus, results should be confirmed by larger, adjusted, real-world studies. The present study was a cross-sectional observational design during hospital stay; hence, it does not provide prognostic data, and patients with complications after hospital discharge were not followed up to assess for long-term clinical outcomes. Regarding RV strain, the septal side may be affected by the left ventricle. Other modalities, e.g., magnetic resonance imaging and single-photon emission computerized tomography, could be used for comparison with other materials regarding RV and infarction size.

## Clinical implication

- Patients with anterior STEMI are at higher risk for RV dysfunction than those with inferior STEMI and were affected by LVEF decline irrespective of the infarction site.
- Increasing interest in RV function assessment techniques in patients with acute STEMI (once considered a forgotten ventricle) is important to improve prognosis, treatment, and outcomes.

## Conclusion

RV dysfunction can occur in patients with no ECG evidence of RV STEMI. RV dysfunction is worse in patients with anterior compared to those with inferior MI. Moreover, RV systolic functions were affected by declined LVEF irrespective of the infarction site. This had potential clinical implications to improve prognosis, treatment, survival rate, and outcomes between both conditions.

## Recommendations

We recommend a follow-up of a large sample study with the change in studied parameters to include RV infarction which requires more studies for its implication.

Future larger multicenter studies are needed to demonstrate clinical outcome data, such as mortality, cardiogenic shock prevalence, and the need for mechanical or inotropic support.

Future mid- and long-term longitudinal studies will need to investigate RV dysfunction as a predictor of outcome in patients with STEMI.

## Data Availability

Our retrospective cross-sectional study data used to support the findings of this study are available from the corresponding author upon request.

## Abbreviations

CA: Coronary angiography
DES: Drug-eluting stent
DM: Diabetes mellitus
ECG: Electrocardiogram
EES: Everolimus-eluting stent
HTN: Hypertension
IHD: Ischemic heart disease
LV: Left ventricle
LVEF: Left ventricular ejection fraction
MI: Myocardial infarction
PCI: Percutaneous coronary intervention
ROC: Receiver operating characteristic curve
RV: Right ventricle
STEMI: ST-elevation myocardial infarction
TTE: Transthoracic echocardiography
